# Communication Skills Assessment and Perceived Barriers of Effective Communication Among Medical Undergraduate Students During Family Adoption Program Visits in a Government Medical College of Assam, India: A Mixed-Method Study

**DOI:** 10.7759/cureus.111634

**Published:** 2026-06-27

**Authors:** Shivangi Priyadarshini, Netri Das

**Affiliations:** 1 Community Medicine, Jorhat Medical College and Hospital, Jorhat, IND; 2 Community Medicine, Lakhimpur Medical College and Hospital, Lakhimpur, IND

**Keywords:** communication skills assessment, community medicine, competency based medical education (cbme), family adoption program(fap), mixed method study

## Abstract

Introduction

The Family Adoption Program (FAP), introduced by the National Medical Commission (NMC) under the competency-based medical education (CBME) curriculum, aims to provide experiential learning opportunities, including effective communication skills among Bachelor of Medicine and Bachelor of Surgery (MBBS) students. This study assessed communication skills among MBBS students during FAP visits, addressed inter-rater variability, and explored perceived barriers to communication while engaging with the adopted families.

Methods

A mixed-methods study was conducted in which the communication skills of 100 MBBS students were assessed using the Gap-Kalamazoo Communication Skill (GKCS) assessment forms (self and faculty versions) from January to June, 2025. Inter-rater variability was addressed using Cohen's Kappa. Also, focus group discussions (FGD) were held among 27 MBBS students to identify barriers to communication during FAP visits.

Results

While students rated their communication skills as "good" across all GKCS competencies, with the highest rating (56%) for opening a discussion, faculty assessed six out of nine competencies as "good," with the highest rating (42%) for building a relationship. Seven out of the nine competencies demonstrated slight agreement beyond chance, with kappa values ranging from 0.038 to 0.150. Some of the perceived barriers of communication faced by the students during FAP visits included inadequate knowledge and understanding, differences in attitude, and linguistic and cultural differences.

Conclusion

The study highlights the need for formal communication skills training and structured feedback among MBBS students. Also, the usage of the local language and prior public awareness about FAP can be recommended to overcome the barriers of communication during FAP visits. The program would be more impactful if one or two families were allotted per student, facilitating meaningful interactions and improving communication skills.

## Introduction

Medical education is predominantly centered on hospital settings, where students are exposed to a limited spectrum of health conditions. Therefore, it demands a broader approach that helps students understand patients not only from the biological point of view but also within the context of society and community [1]. Studies across the world have highlighted the importance of community-based medical education, like Longitudinal Integrated Clerkships in Australia and Canada, where students develop good communication as well as clinical skills [2]. Similarly, in India, the Village Adoption Scheme (VAS) with its social service camp conducted by Mahatma Gandhi Institute of Medical Sciences (MGIMS), Sewagram, shows successful participation of medical students in building an interface between the community and healthcare [3]. The National Medical Commission (NMC) introduced the Family Adoption Program (FAP) under the competency-based medical education (CBME) curriculum for undergraduate (UG) students from the 2021-2022 academic year to enhance equity in health while offering experiential learning opportunities to Indian medical graduates. The program has set student targets such as learning communication skills, health programs, data analysis, understanding rural dynamics, along with disease identification and improvement in the overall quality of the lives of adopted families [4-6].

One of the significant roles of an Indian medical graduate is that of an effective communicator with patients, families, colleagues, and the community at large. Therefore, self-awareness regarding their communication skills is important for building a healthy doctor-patient relationship in the future [4]. Also, feedback is essential during the initial stage of its implementation for immediate corrections, and measures proposed by the participants themselves could be utilized to make FAP more impactful [3,7,8]. It is the students whose perspective can throw light on the operational feasibility and the challenges encountered. Moreover, as FAP is a relatively novel concept, this study has the potential to contribute valuable evidence to the existing research work. Hence, the present study was undertaken to assess the communication skills of MBBS students during FAP visits using Gap-Kalamazoo Communication Skill (GKCS) assessment form (self and faculty versions) and to determine if there existed any inter-rater variability while assessing the same. This study also aims to explore the perceived barriers to communication among the Bachelor of Medicine and Bachelor of Surgery (MBBS) students during FAP visits.

## Materials and methods

This institution-based cross-sectional study, using a convergent parallel approach (a type of mixed-method study), was conducted from January to June 2025 among MBBS students in phases 1 (n=121), 2 (n=131), and 3 part 1 (n=121) at a government medical college in Assam. Quantitative and qualitative data were analyzed independently. Narrative integration was achieved at the interpretation stage using a contiguous approach, wherein findings from both aspects were presented in distinct segments and interpreted to develop a comprehensive understanding of the matter and enhance complementarity.

For the quantitative aspect, the sample size was calculated by using Cochran’s formula: (z^2^pq)/d^2^. By taking prevalence as 52.73% [9] and 10% as an absolute error, (3.84*0.527*0.473)/0.01=96. The sample size was rounded off to 100. The sampling technique involved was convenience sampling. Simultaneously, a phenomenological qualitative study was conducted among 27 MBBS students in the form of three focus group discussions (FGDs), selection for which was done by a purposive type of non-probability sampling. For both quantitative and qualitative aspects, MBBS students who were available at the time of data collection were included in the study.

Data collection

Quantitative Aspect

Gap-Kalamazoo Communication Skills assessment form (self-version) containing nine competencies with a five-point Likert scale for each competency was distributed among the study participants while they were on FAP visit, and were asked to rate themselves. Similarly, the faculty version of the GKCS assessment form was used by the faculty members of the Department of Community Medicine to rate them based on their interaction with the adopted families. No formal faculty selection procedure was undertaken as students were assessed during a routinely conducted educational activity of scheduled FAP visits. The nine competencies included - builds a relationship, opens a discussion, gathers information, understands the family’s perspective, shares information, reaches an agreement, provides closure, demonstrates empathy, and communicates accurate information (Appendix) [10]. Keeping an eye on the quality of research, not more than 10 students were rated in a day.

Qualitative Aspect

Students from each phase, interested in expressing themselves, were purposively approached. Three FGDs were held in person, and the audio was recorded throughout. There were 27 study participants: phase 1 (nine students), phase 2 (10 students), and phase 3 [part 1] (eight students), assuring representativeness from each phase exposed to FAP as per Graduate Medical Education Regulations (GMER) 2022 and 2023 guidelines. A FGD guide was used to carry forward the discussion on barriers to communication while engaging with the adopted families.

Data analysis

Quantitative Aspect

Data was compiled in Microsoft Excel 2021 (Microsoft Corporation, Redmond, USA) and summarized as counts and percentages. Also, wherever relevant, graphical presentation was done using multiple/clustered bar diagrams. Inter-rater variability in the assessment was addressed using Cohen’s Kappa [11].

Methodological Consideration

Cohen's kappa was selected because the communication skill assessment scores were analyzed as categorical ratings across predefined Likert-scale categories rather than as continuous measurements. In the present study, student self-ratings and faculty ratings represent assessments from two distinct perspectives rather than equivalent interchangeable raters. Therefore, the focus was on agreement between rating categories rather than measuring the consistency of interchangeable observers. For this reason, Cohen's kappa was considered.

All response categories were treated as distinct categories. The present study attempted to determine whether the student and faculty assigned the same rating category. The magnitude of disagreement between adjacent and distant categories was not intended to be incorporated into the analysis. Accordingly, Cohen's kappa was applied.

Qualitative Aspect

All recordings were manually transcribed in English. Verbatim statements were repeatedly heard to derive codes from them. Each line or segment of a line that contributes to the concerned objective was identified with a code. Similar codes were categorized under specific sub-themes, which were further categorized under broader themes, and hence, inductive thematic analysis was done. Data collection and analysis were conducted simultaneously. Although the number of FGDs was not previously fixed, three FGDs (one representing each phase) revealed enough information on the matter. By the time analysis of the third FGD was over, data saturation was considered achieved when no new codes emerged from successive verbatims, and the existing thematic framework adequately captured participants' perspectives.

Coding validation process: There was involvement of two co-coders who first grasped the idea of the broader picture through initial coding. Then, line-by-line coding was done in the subsequent rounds, and ultimately, categories were finalized based on the codes. The credibility of the coding was further enhanced through member checking (participant validation), whereby participants were allowed to review and confirm the interpretation of the findings.

Ethical consideration and informed consent

Ethical clearance bearing the number SMEJ/JMCH/MEU/841/Pt-III/2023/4816 was obtained from the Institutional Ethics Committee, Human (IEC, H) Jorhat Medical College and Hospital (JMCH), Jorhat, before beginning the study. Permission to use the Gap-Kalamazoo Communication Skills Assessment Form (faculty and self-assessment versions) was obtained. 

Written informed consent was taken from each study participant. In addition, permission was taken before recording the audio during the FGDs.

## Results

Out of the total 373 UG students exposed to FAP as per GMER 2022 and 2023 guidelines, 100 were included in the study as per the inclusion criteria for communication skill assessment, and 27 students were considered for three FGDs.

The median age of study participants was 22 years (interquartile range: 21-23). The gender distribution of the study participants showed that 54% were male and 46% were female. There was an equal distribution of study participants by residence, with 50% in rural areas and 50% in urban areas. 

**Figure 1:**
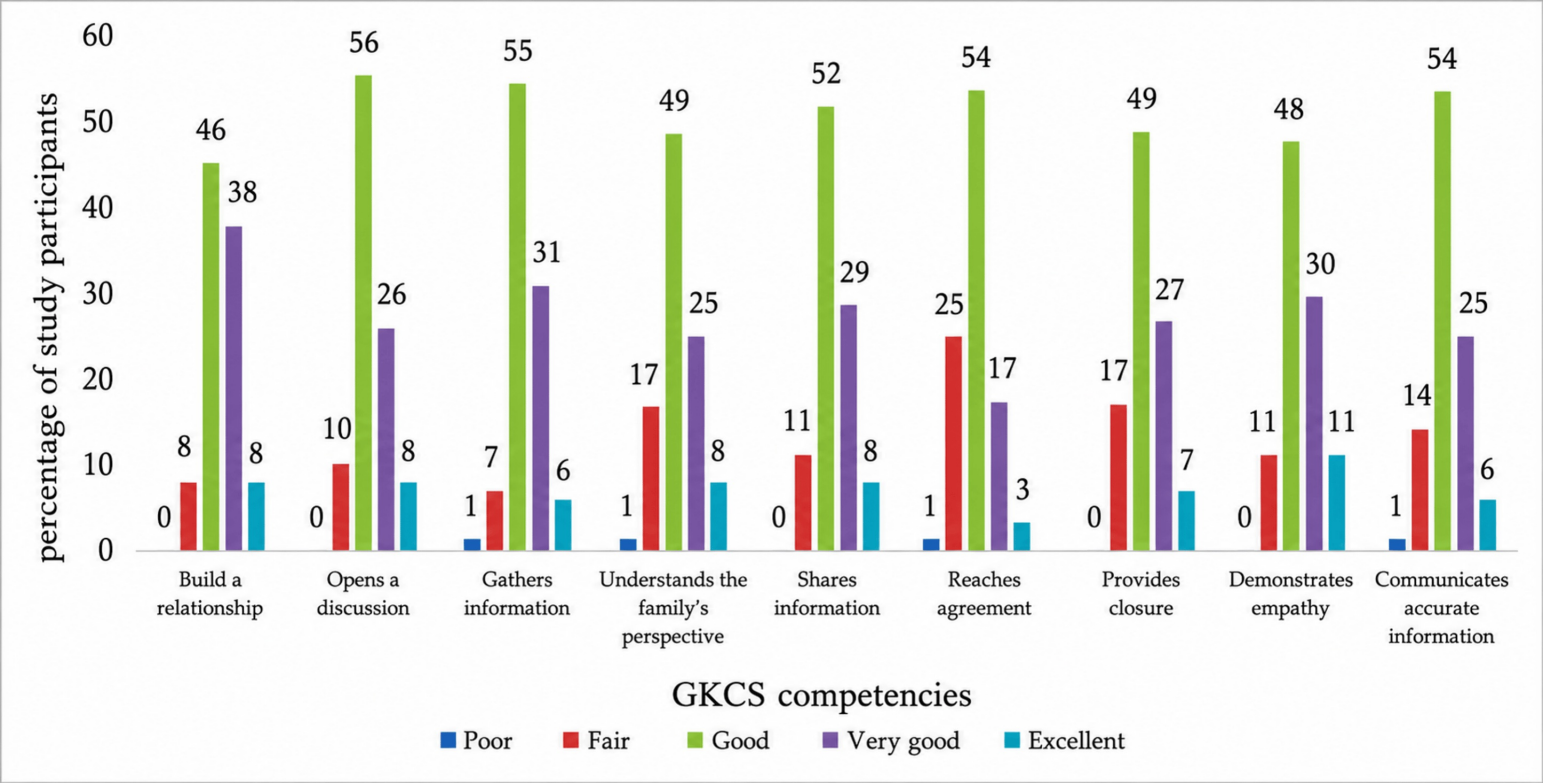
Multiple bar diagram showing communication skill assessment using GKCS assessment form (self version) GKCS: Gap-Kalamazoo Communication Skills

As shown in Figure 1, the majority of the students rated their communication skills as ‘good’ across all GKCS competencies, with the highest in the competency of opening a discussion (56%). ‘Excellent’ rating was present across all competencies, with the highest in the competency - demonstrates empathy (11%). ‘Poor’ rating was negligible, reflecting their overall positive self-perception of communication skills.

**Figure 2:**
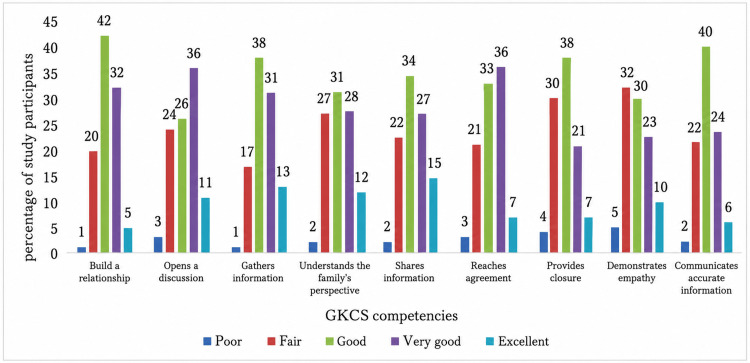
Multiple bar diagram showing communication skill assessment using GKCS assessment form (faculty version) GKCS: Gap-Kalamazoo Communication Skills

Figure 2 shows the faculty version of the GKCS assessment in which ‘good’ rating was the highest for the competency - build a relationship (42%). ‘Excellent’ rating was present across all competencies, with the highest in the competency - shares information (15%). Also, a minimal number of study participants were rated ‘poor’ across various competencies, with the highest in the competency - demonstrates empathy at 5%.

As shown in Table 1, inter-rater variability was addressed using Cohen’s Kappa statistics across the nine GKCS competencies. Seven out of nine competencies demonstrated slight agreement beyond chance, with kappa values ranging from 0.038 to 0.150. Overall, the level of agreement across most GKCS domains was low, highlighting inter-rater variability.

**Table 1:** Inter-rater variability in communication skills assessment using Cohen's Kappa

Gap Kalamazoo Competencies	Observed agreement (p₀)	Chance agreement (pₑ)	Cohen Kappa k=(p₀-pₑ)/(1-pₑ)	Inference
Builds a relationship	0.39	0.3348	0.083	Slight agreement
Opens a discussion	0.34	0.272	0.093	Slight agreement
Gathers information	0.35	0.3249	0.073	Slight agreement
Understands the family’s perspective	0.34	0.2776	0.086	Slight agreement
Shares information	0.26	0.2913	-0.044	No agreement
Reaches agreement	0.29	0.2918	-0.003	No agreement
Provides closure	0.35	0.2988	0.073	Slight agreement
Demonstrates empathy	0.37	0.2588	0.15	Slight agreement
Communicates accurate information	0.34	0.3139	0.038	Slight agreement

Table 2 shows the findings of the qualitative aspects of the study, where broad themes emerged in decreasing order of frequency: difference in attitude, conflict in interpersonal relationships, inadequate knowledge and understanding, linguistic barrier, and cultural differences. The students themselves suggested prior public awareness regarding FAP, use of local language and many more measures to tackle the barriers of communication while engaging with the adopted families. The quantitative data were supplemented by the qualitative themes to portray the complete picture.

**Table 2:** Theme generation from verbatims using inductive thematic analysis The number within brackets beside each theme denotes the number of times a particular theme emerged from the verbatims

Representative Verbatims	Codes	Sub-themes	Themes
“The concept of family adoption program was also very new to me. I was not being able to tell them exactly what they will get.”, "They (adopted family members) ask me what to do but deep inside even I don't know what to."	Confusion of role/difficult to explain/not fully physicians	Confusion of students’ role	Inadequate knowledge and understanding (12)
“You don’t have to teach us. You’re a little girl.", "They (adopted family members) were not totally confident about us."	Doubt about students’ knowledge/student might not know much	Skeptical about students’ competence	
"What contraceptives do you use? Now this is awkward for the student to ask and awkward for the family member to reply.”, "Hesitant to provide any detail."	Non-cooperative/hesitation to interact	Resistance from families	Difference in attitude (23)
“How many times do you come?? how many times do you ask questions?”, "Families were annoyed by our repeated visits."	Annoyance due to repeated visits	Annoyance from families	
“In FAP, we get a family regardless of their needs…we just impose on them.”, "Those who actually need family adoption, they aren't getting it."	Perceived imposition upon families	Burden to families	
"They (referring to batchmates) were being treated like how salesmen on visit are treated.”, "Didn't really respond."	Lack of respect/feeling ignored	Compromised dignity	Conflict in interpersonal relationships (15)
"Come next time.", "I was not welcomed like I had expected."	Feeling of not being welcomed		
"I remember one time I was asking them if they had their adhar card…..felt the need to give me a lesson on the ongoing frauds.", "Not much interested"	Lack of trust/interest in FAP	Trust issues	
“…they have trust issues regarding the fact that I’m from outside. ", "They didn't want to trust us."	Lack of trust in students		
“Speaking in Hindi isn’t very trust worthy for them.", "Language barrier is the main issue."	Difference in language	Language difference	Linguistic barrier (6)
“Till now they are not fine with me entering their kitchen. I don’t know why……...a difficult time for me to create the connection.”	Restriction in viewing the kitchen	Social/religious restrictions	Cultural differences (3)
“…. Now I have to do the puja and all. That’s why I’m not going to do any kind of interaction.”	No interaction before rituals		
"So the husband says that I’m not going to do any tests, not even my wife going to do anything."	Husband decides for wife		

## Discussion

The key findings of this study were that most students rated their communication skills as ‘good,’ followed by ‘very good’ ratings in almost all the GKCS competencies, with significantly lower percentages of ‘poor’ and ‘excellent’ ratings. A similar finding was observed in a study done by Baruah et al. from Assam, where 52.73% of the students felt that they had gained communication skills through FAP [9]. In a study conducted by Chepuru et al., 58.9% of the students agreed in a five-point Likert scale that FAP helps learn communication skills [12]. Another study conducted by Ganganahalli et al. in Karnataka found 75.7% of the students agreed to the fact that FAP has enhanced their communication skills on a three-point Likert scale [13]. On the contrary, similar research carried out by Gurav et al. found that 73.71% of the students strongly agreed on a five-point Likert scale that FAP is important for communication skills improvement [14]. In a similar study conducted by Vairavasolai et al. in Tamil Nadu, 93% of the students responded positively in a dichotomous rating scale [5]. Now, the disparity in the later studies might be due to the use of different rating scales. Also, there could be a difference in self-confidence when assessing their own communication skills among the students.

Upon rating the students using the faculty version of the GKCS assessment form, they were rated ‘good’ in six out of the total nine competencies. In fact, the contribution of the ‘fair’ rating was highest for the competency - demonstrates empathy (32%). On the other hand, up to 15% of the students were rated ‘excellent’ in the competency - shares information. Also, students were rated ‘poor’ in various competencies, with the highest in the competency of sharing information at 15%. To address this inter-rater variability, whether the difference was due to chance or in reality, Cohen’s kappa was applied. It was found that there was only slight agreement between the raters across seven out of the nine competencies and absolutely no agreement in the remaining two. This shows that there exists inter-rater variability. This variation has resulted due to a variety of probable reasons. One of them could be that students wanted to place themselves in a neutral position by rating ‘good’ (three points in a five-point Likert scale). They might have a perception that rating ‘poor’ (one point in a five-point Likert scale) would reveal their weakness and that rating ‘excellent’ (five points in a five-point Likert scale) would make them appear overconfident. Another possibility might be a lack of self-awareness. The observer’s rating was more critical, and hence the overall percentage of ‘Good’ rating across various domains was lower than that observed in their self-assessment. This can also be addressed by the difference in Likert scale interpretation between the raters. What is ‘good’ for one might be ‘fair’ for another.

From the FGDs, five themes signifying the communication barriers emerged, as shown in Table 2. Certain students revealed that they themselves weren’t clear about their role in FAP. Also, according to them, some adoptive families were doubtful about the students’ knowledge. There seems to be a gap in knowledge and understanding, and hence in communication. Some students expressed about the resistance and annoyance at the adopted families. Language barrier seemed to play an important role too, as there were several students who weren’t from Assam. Langde et al. from Maharashtra also cited language barrier and reluctant cooperation as pertinent issues [15]. Also, a study conducted by Yalamanchili et al., one of the themes mentioned was about questionable acceptance of students by the adopted families [7]. A lot of students also pointed out the conflicts in interpersonal relationships while communicating with the adopted families, where a lack of trust and respect were major hindrances to effective communication. Also, there were religious and social restrictions that stood as barriers to communication. A study conducted by Shree et al. in Mysuru also depicted similar findings [6].

Limitations

There were certain limitations to the study in terms of interpretation and extrapolation. The study was based on self-reporting and individual perception rather than objective measurements. The advantages of a longitudinal study and hence follow-up couldn't be availed as it was a cross-sectional study. Pertaining to the feasibility of the study at the field level, the convenience sampling technique was used, while the communication skills assessment of MBBS students during the FAP visit doesn’t obey the law of probability. The fact that students willing to express themselves were purposively chosen for the qualitative aspect, may have a possibility of introducing selection bias to the study. The current study considers only one medical college; as a result, the findings may not portray a holistic picture of FAP implementation in the entire country. Consequently, the findings may not be representative of other medical institutions and should be interpreted cautiously.

## Conclusions

From the present study, it is revealed that self-awareness of students regarding their communication skills is needed, and the inter-rater variability points towards the difference in self-assessment and faculty assessment, but does not justify the superiority of one rater over another. Hence, formal training and structured feedback by trained persons exclusively for communication skill development is required. To reduce the barriers of communication, usage of the local language, prior public awareness about FAP, frequent health camps, and mutually convenient timings should be encouraged. FAP has the scope to be more impactful if one or two families are allotted per student, thereby facilitating meaningful interactions and improving communication skills. Last but not least, for effective communication to begin and sustain, mutual respect for one another acts as the driving force. 

## Appendices

Appendix 

**Table 3:** Nine competencies of GKCS assessment form (self-version) and their key components GKCS: Gap-Kalamazoo Communication Skills

Sl. No.	Communication Competency	Key Components							
1	Builds a Relationship	Greets and shows interest in the patient and family; uses words expressing care and concern; demonstrates caring through tone, pace, eye contact, and posture; responds to patient and family ideas and feelings.							
2	Opens the Discussion	Allows patient and family to complete opening statements without interruption; asks 'Is there anything else?'; explains or negotiates the agenda for the visit.							
3	Gathers Information	Uses open-ended questions; clarifies details with specific or yes/no questions; summarizes information and allows corrections; transitions effectively to additional questions.							
4	Understands the Patient's and Family's Perspective	Explores life events, circumstances, and social influences affecting health; elicits beliefs, concerns, and expectations regarding illness and treatment.							
5	Shares Information	Assesses understanding and desire for information; explains in understandable language; invites questions from the family.							
6	Reaches Agreement	Involves family in decisions; checks mutual understanding of diagnostic and treatment plans; assesses acceptability of plans; identifies additional resources when appropriate.							
7	Provides Closure	Invites final questions or concerns; summarizes key points; clarifies future follow-up; provides contact information if needed; acknowledges patient and family and closes the interview.							
8	Demonstrates Empathy	Maintains appropriate demeanor; shows compassion and concern; identifies and validates emotions; responds appropriately to emotional cues.							
9	Communicates Accurate Information	Accurately conveys seriousness of condition; considers input from other clinicians; explains expected disease course; presents future care options clearly; provides sufficient information for informed decision-making.							
